# Evaluating contribution of ionic, osmotic and oxidative stress components towards salinity tolerance in barley

**DOI:** 10.1186/1471-2229-14-113

**Published:** 2014-04-28

**Authors:** Getnet Dino Adem, Stuart J Roy, Meixue Zhou, John P Bowman, Sergey Shabala

**Affiliations:** 1 School of Land and Food, University of Tasmania, Private Bag 54, Hobart Tas 7001, Australia; 2 Australian Centre for Plant Functional Genomics, Private Mail Bag 1, Glen Osmond SA 5064, Australia; 3 University of Adelaide, Private Mail Bag 1, Glen Osmond SA 5064, Australia

**Keywords:** Stomatal conductance, Sodium sequestration, Potassium retention, Membrane potential, Tissue specific responses, H^+^-ATPase, Reactive oxygen species, Cytosolic ion homeostasis

## Abstract

**Background:**

Salinity tolerance is a physiologically multi-faceted trait attributed to multiple mechanisms. Three barley (*Hordeum vulgare*) varieties contrasting in their salinity tolerance were used to assess the relative contribution of ionic, osmotic and oxidative stress components towards overall salinity stress tolerance in this species, both at the whole-plant and cellular levels. In addition, transcriptional changes in the gene expression profile were studied for key genes mediating plant ionic and oxidative homeostasis (*NHX*; *RBOH*; *SOD*; *AHA* and *GORK*), to compare a contribution of transcriptional and post-translational factors towards the specific components of salinity tolerance.

**Results:**

Our major findings are two-fold. First, plant tissue tolerance was a dominating component that has determined the overall plant responses to salinity, with root K^+^ retention ability and reduced sensitivity to stress-induced hydroxyl radical production being the main contributing tolerance mechanisms. Second, it was not possible to infer which cultivars were salinity tolerant based solely on expression profiling of candidate genes at one specific time point. For the genes studied and the time point selected that transcriptional changes in the expression of these specific genes had a small role for barley’s adaptive responses to salinity.

**Conclusions:**

For better tissue tolerance, sodium sequestration, K^+^ retention and resistance to oxidative stress all appeared to be crucial. Because these traits are highly interrelated, it is suggested that a major progress in crop breeding for salinity tolerance can be achieved only if these complementary traits are targeted at the same time. This study also highlights the essentiality of post translational modifications in plant adaptive responses to salinity.

## Background

The world food supply is endangered by salinity exacerbated by global environmental warming. Agricultural production is already seriously affected by increasing salinity, with estimated economic penalties being in excess of $12B [1,2]. Creating salt tolerant crop germplasm is, therefore, becoming an urgent imperative [3,4].

Salinity tolerance in crops is a physiologically multi-faceted trait and is attributed to multiple mechanisms. The key ones include improved osmotic adjustment; minimising Na^+^ uptake by roots and/or increasing Na^+^ efflux back to the soil; intracellular Na^+^ sequestration; potassium retention in the cytosol; tissue-specific Na^+^ sequestration; control of xylem ion loading; excluding Na^+^ from the shoot; and oxidative stress tolerance [5-7]. These numerous mechanisms are usually grouped into three major clusters: (i) osmotolerance; (ii) sodium exclusion mechanisms; and (iii) tissue tolerance mechanisms [6]. Despite the significant progress that has been made in elucidating specific details of each of these mechanisms, the relative contribution of the above components to overall salinity tolerance remains unclear, prompting numerous attempts to overcome the issue by modifying the expression level or function of specific genes by molecular means [8,9].

Crop osmotolerance has long been attributed to a plant’s ability to increase *de novo* synthesis of compatible solutes [10,11]. Accordingly, efforts have been made to increase the expression of compatible solute associated genes, most significantly those that catalyse the production of glycine betaine including betaine aldehyde dehydrogenase, encoded by the gene *betB*[12,13], choline dehydrogenase (CDH) encoded by *betA*[14] and choline oxidase *codA*[15]. Despite these genes being transformed into a large number of species, the success in providing improved crops to the farmers field via this avenue has been very limited [16,17].

Another major component of salinity tolerance often targeted in transgenic plants is Na^+^ exclusion by minimising Na^+^ uptake by the root. This component comes to play by the *SOS1* (plasma membrane Na^+^/H^+^ exchanger) along with interacting and phosphorylating proteins *SOS3* and *SOS2* forming a Ca^2+^-dependent signalling cascade [18,19]. This Na^+^ exclusion process is energised by the plasma membrane H^+^-ATPase [20]. Over-expressing *SOS1* Na^+^ exclusion gene or its homologues has been attempted in several species such as Arabidopsis [18] or tobacco [21]. However, when Na^+^ is extruded to the medium by this mechanism, it further increases the osmotic and ionic imbalance that in turn causes the stress to be aggravated. Therefore, such a strategy can only be used as an interim solution and cannot, by itself, confer long term salinity stress tolerance under field conditions. Other mechanisms contributing to restricted Na^+^ accumulation in the shoot include reduced Na^+^ loading into the xylem [6,22] and increased Na^+^ retrieval from the xylem [23], with a recent study by Munns et al. [3] showing that the presence of *TmHKT1;5-A* significantly reduced leaf Na^+^ content and increased durum wheat grain yield by 25% compared to near-isogenic lines lacking a Nax2 locus, expressing this gene. Sodium retrieval from the shoot via its recirculation to the root via phloem is another contributing mechanism [24], this process is also believed to be mediated by HKT transporters [25]. However, it has been argued that excluding Na^+^ from the xylem may not be a plausible mechanism at all times [26], as Na^+^ can be used as a cheap osmoticum in species possessing high tissue tolerance mechanisms. Indeed barley over-expressing the *HKT* subfamily 2 gene, *HvHKT2;1*, had higher xylem and leaf Na^+^ content in saline-grown plants and was correlated with increased salt tolerance [27].

The third component of salinity tolerance is tissue tolerance, e.g. the ability of plants to safely handle large amounts of sodium accumulated in plant tissues, without any detrimental effects to cell metabolism. Such tissue tolerance can be achieved by efficient Na^+^ sequestration away from the cytosol (where it can affect the metabolic processes of the cell) into the vacuole. This is achieved by operation of the tonoplast Na^+^/H^+^ exchanger [28-30] that is energised by the vacuolar H^+^-ATPase (V-ATPase, E.C. 3.6.1.3) and pyrophosphatase (V-PPase, E.C. 3.6.1.1.) [31]. The *Arabidopsis thaliana* Na^+^/H^+^ antiporter gene (*AtNHX1*) was the first plant *NHX* homolog to be cloned [30]. There are six isoforms of *AtNHX* in Arabidopsis with *AtNHX1* and *AtNHX2* highly expressed in many tissues, while *AtNHX3* and *AtNHX4* are exclusively expressed in flowers and roots [32,33]. Recently, evidence has emerged suggesting that *NHX1* proteins may operate as K^+^/H^+^ exchangers, and that their major role is not Na^+^ transport but regulation of vacuolar K^+^ content [34,35].

Vacuolar Na^+^ sequestration is important but not the only mechanism contributing to tissue tolerance. The ability to retain K^+^ in cells has recently emerged as an important component of this trait, in both root [36-39] and leaf [40] tissues. Strong correlation between K^+^ retention ability and plant overall salinity tolerance has been shown in some species [37,38,41] and attributed to the importance of high cytolosolic K^+^ to suppress activity of caspase-like proteolytic and endonucleolytic enzymes triggering programmed cell death in salt-affected cells [42,43]. High cytosolic K^+^ is also required to maintain metabolic processes such as protein synthesis by enabling tRNA binding to ribosomes [44] Also contributing to plant tissue tolerance is reactive oxygen species (ROS) detoxification. It has been shown that significant amounts of ROS are generated in salt-affected plant tissues in both roots and leaves [45,46], and the causal link between salinity and oxidative stress signalling and reactive species detoxification is becoming evident [47]. At the same time, attempts to link plants salinity tolerance with the level of antioxidant activity in their tissues appear to be problematic, with reports being highly controversial and ranging from positive to either negative or no correlations with salinity stress tolerance [47,48].

To the best of our knowledge, only one comprehensive attempt to separate the relative contribution of each of above three major components of salt stress was reported in the literature. Using sophisticated whole-plant imaging facilities to determine the area of healthy leaf and the area of senescing leaf in several einkorn wheat accessions, Rajendran et al. [49] has reported that the most tolerant genotype (judged by relative growth rate under saline conditions) was the *Triticum monococcum* accession, AUS 18755–4 which was not the best performing in any of the above three major mechanisms contributing to salinity tolerance, namely Na^+^ exclusion, osmotic tolerance, and tissue tolerance, compared to the other eleven accessions studied. On the contrary, this variety had an excellent osmotic tolerance (indexed as 0.95 out of 1) and good tissue tolerance but had rather poor ability to exclude Na^+^ (indexed as only 0.17). However, given the indirect methods of assessment used (e.g. whole-plant phenotyping) and the fact that this work has been conducted on salt-sensitive species (wheat), it remains to be answered to what extent these conclusions can be extrapolated to other species.

Barley is one of the most important cereal crops in the world. While being generally classified as relatively salt tolerant (Maas & Grieve 1984), barley germplasm show a great extent of variability in salinity stress tolerance [38,50]. We used this opportunity to examine the salt tolerance mechanisms at the post-translational level and compared it with changes observed at the transcript level. Unlike Rajendran et al. [49], our assay has been conducted not only at the whole plant but also at the cellular level (using the non-invasive microelctrode ion flux measuring (MIFE) technique). We also aimed to compare contribution of transcriptional and post-translational factors to the specific components of the overall salinity tolerance. Our results indicate that root K^+^ retention ability and increased tolerance to ROS damage were the main contributing tolerance mechanisms. These traits were pronounced at a post translational but not a transcriptional level. The overall poor correlation of the change in transcript levels of selected genes in relation to post translational/functional response, demonstrates the importance of post translational modifications *in planta*.

## Methods

### Plant materials and growth conditions

#### Glasshouse experiments

Three barley (*Hordium vulgare* L.) cultivars - Numar (salt tolerant), Naso Nijo (salt sensitive) and Golden Promise (intermediate salinity tolerance) – were used in experiments. Seeds were obtained from the Tasmanian Institute of Agriculture (TIA) and University of Adelaide Waite Barley Breeders. Seeds were planted into 2 L plastic pots containing 70% composted pine bark; 20% coarse sand; 10% sphagnum peat; Limil at 1.8 kg/m^3^, dolomite at 1.8 kg/m^3^). The plant nutrient balance was maintained by adding the slow release Osmocote Plus™ fertilizer (at 6 kg/m^3^), plus ferrous sulphate (at 500 g/m^3^) [51]. Two levels of NaCl (0 and 150 mM) were applied in five replications and eight plants were grown in each 2 L pot. The plants were grown from seed under controlled greenhouse conditions (temperature between 19 and 26°C; day length, 12 h; average humidity ~65%) at the University of Tasmania between March 2012 and April 2012. The plants were irrigated with salt free water until seedling establishment (approx. for one week) and then after, the plants were irrigated with the respective salt treatments for four weeks.

#### Electrophysiological experiments

Seeds were surface sterilized by 1.5% (w/v) NaClO and rinsed well with distilled water several times. The seeds were germinated and grown for 3 days in an aerated hydroponic solution containing 0.5 mM KCl and 0.1 mM CaCl_2_ in a dark growth chamber at 24 ± 1°C as described elsewhere [26,52]. Plants were used for measurements when their roots were 60 to 70 mm long.

#### Hydroponic experiments

Barley plants were grown from seeds in 1 L plastic pot in 25% strength of modified Hoagland solution [53] for 3 days where the first leaf fully emerged. To salt stress the plants, 100 mM of NaCl was then added to the hydroponics solution, and plants were grown for additional 8 days. Root and shoot tissues were sub-sampled on days 1, 2, 4, and 8 after salt application. Control plants were grown in 25% strength of Hoagland solution for the entire duration of experiment.

### Whole-plant agronomical and physiological characteristics

Shoot fresh (FW) and dry (DW) weight were measured, and relative water content calculated as RWC = (FW-DW)/FW. Before harvesting, leaf chlorophyll content was measured as a SPAD index using a Minolta Chlorophyll Meter SPAD-502 (Konica Minolta, Osaka, Japan) on the third true leaf, at a position about one quarter of the length of the leaf from the leaf tip. Stomatal conductance (Gs) was also measured on the same leaf using a Decagon leaf porometer (Decagon Devices Inc., WA, Australia), under constant light conditions (artificial light of 150 μmol m^−2^ s-^1^). The number of necrotic leaves was also counted at harvest.

### Tissue sap ion content

Na^+^ and K^+^ content in plant tissues was determined by the freeze-thaw method essentially as described elsewhere [54]. In brief, roots of hydroponically-grown plants were quickly rinsed in 10 mM CaCl_2_ to remove apoplastic Na^+^, blotted dry and then placed into 1.5 mL microfuge tubes and stored at −20°C. Shoot samples were harvested at the same time and also frozen at −20°C. The frozen samples were thawed and the sap squeezed from the tissue using a pointed glass rod. The sap samples were diluted × 100 times with distilled water, and K^+^ and Na^+^ content of the sap determined using a flame photometer (MODEL PFP7 Flame photometer, JENWAY, Bibby Scientific Ltd, UK).

### Non-invasive ion flux measurements

Net fluxes of K^+^ and H^+^ were measured using non-invasive ion measurement technique, MIFE (University of Tasmania, Hobart, Australia) as described in our previous publications [26,52]. In brief, borosilicate glass microelectrodes with the tip diameter of 2–3 μm were pulled, silanised with tributylchlorosilane (Fluka, Catalogue no. 90796), and then filled with appropriate back-filling solution. Electrode tips were then filled with an appropriate Liquid Ion Exchanger (LIX) (Fluka Catalogue no. 60031 for K^+^; 95297 for H^+^). Microelectrodes were calibrated in a set of pH and K^+^ standards before and after use. The electrodes were mounted on a 3D-micromanipulator (MMT-5, Narishige, Tokyo, Japan) and the tips of the electrodes were drawn close to each other and positioned 40 μm above the root surface. While measuring, the electrodes were moving between two positions (40 and 80 μm) in a 10 s square-wave manner. The CHART software records the potential difference between these two positions and converted it to electrochemical potential difference considering the Nernst slope value obtained during calibration. These potential difference values were converted into ion flux using MIFEFLUX software utilising cylindrical diffusion geometry (see Newman, 2001). Ion fluxes were measured from excised root segments of 3 to 4 day old seedlings from elongation (~2 mm from the tip) and mature (~10 mm) root zones. Root segments were placed in 10 mL Perspex measuring chamber filled with basic salt medium (BSM; 0.5 KCl mM, 0.1 CaCl_2_ mM, pH 5.7 unbuffered) and allowed to equilibrate for ~ 30 minutes. Steady- state ion fluxes were then recorded for 5–10 min, and then the treatment (either 100 mM NaCl – for salinity stress; or 0.3 mM CuCl_2_ + 1 mM ascorbate – for ROS stress) was administered.

### Gene expression studies

Barley cultivars were grown hydroponically until the third leaf was fully emerged (plants were approximately 15 days). The growth solution was changed every seven days. Plants were treated with 100 mM NaCl for 48 hours, their roots and leaves harvested, and snap frozen with liquid nitrogen. Total RNA was extracted following the method of P Chomczynski [55], using TRIzol reagent (Invitrogen, Carlsbad, CA, USA). Ambion’s DNA-free (Madison, WI, USA) reagent was used to remove contamination of genomic DNA. To synthesise cDNA, Invitrogen’s superscript III Reverse Transcriptase kit with an oligo(dT)_20_ primer was used, following the manufacturer’s instructions. Quantitative Real-time PCR was performed as described in [56] using a RG6000 Rotor-Gene real time thermal cycler (Corbett Research, Sydney) and SYBR® green PCR reagent (Bio-Rad Laboratories, Gladesville). Primers were designed to determine the expression of a number of key genes involved in Na^+^ compartmentation and ROS detoxification and included members of the *NHX*, *AHA*, *RBoHF*, *SOD* and *GORK* family of genes. Primer sequences can be found in Additional file 1: Table S1. Normalization of the test gene transcript was relative to the control gene (*GAPdH2*).

### Statistical analysis

All the values in this manuscript are presented as mean value ± SE. For mean comparison and statistical significant level pairwise t-test in all possible combination of the treatments was employed using SPSS software version 20 (IBM support portal, USA).

## Results

### Whole-plant physiological responses

**Figure 1:**
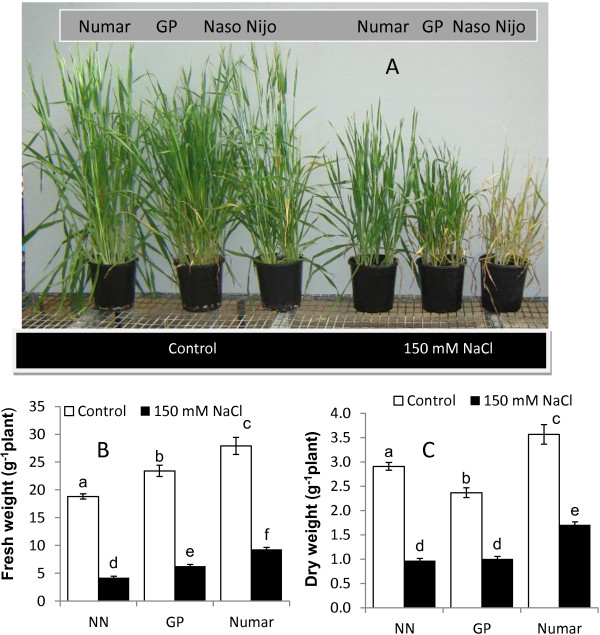
**Growth responses and agronomical characteristics of three barley cultivars (Numar, Golden Promise, and Naso Nijo) treated with 150 mM NaCl for 4 weeks. A **– plant phenotype under control and salt conditions; **B** – shoot fresh weight; **C** – shoot dry weight. Open bar - control; closed bar - salt. Mean ± SE (n = 30).

In the glasshouse, salinity stress significantly affected plant growth and biomass production (Figure 1A), resulting in a three- and five-fold reduction in the fresh weight (FW) in the tolerant variety Numar and the sensitive cultivar Naso Nijo (NN), respectively (Figure 1B). The cultivar Golden Promise (GP) displayed an intermediate salinity tolerance with a 4-fold FW reduction in biomass production under salt stress. All the differences were significant at P < 0.001.

**Figure 2:**
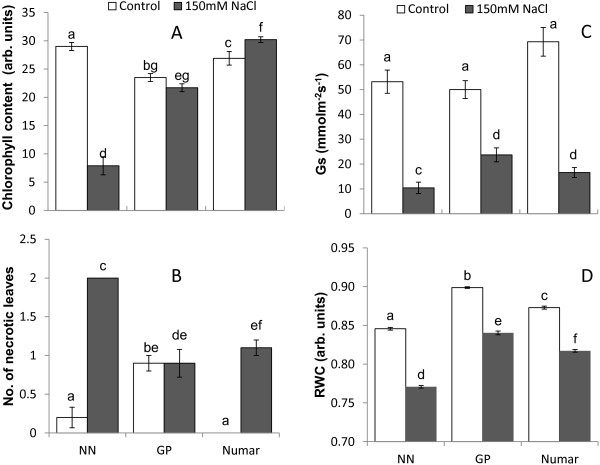
**Whole-plant physiological characteristics in control- and salt-grown (150 mM NaCl for 4 weeks) plants. A** - chlorophyll content (SPAD readings); **B** - number of necrotic leaves; **C** – stomatal conductance (Gs); **D** – relative water content. Open bar - control; closed bar - salt. Mean ± SE (n = 10 and 30 for **A**-**C** and **D**, respectively).

Four weeks of salinity stress also resulted in a reductions in leaf chlorophyll content (Figure 2A). This reduction in chlorophyll varied significantly among cultivars, with the salt sensitive NN exhibiting the highest reduction in chlorophyll content (a 4-fold reduction from 29 ± 0.7 to 7.9 ± 1.6 arbitrary units; significant at P < 0.001). The salt-tolerant cultivar Numar, however, increased its leaf chlorophyll content by ~ 10% (significant at P < 0.05), while the intermediate salt tolerant GP showed no significant reduction in chlorophyll content. The chlorophyll content of the salt grown Numar leaves was 4-fold greater than that measured in salt stressed NN leaves (all the differences are significant at P < 0.01: Figure 2A). The salt-sensitive NN had twice as many necrotic leaves as Numar and GP (Figure 2B).

Salinity stress also resulted in a significant (P < 0.01) decline in stomatal conductance (Gs; Figure 2C) and shoot water content (RWC; Figure 2D). The highest decline in Gs was measured in the sensitive cultivar NN (a five-fold reduction from 53.2 ± 4.7 to 10.4 ± 2.3 mmol m^−2^ s^−1^). Surprisingly, the intermediate cultivar GP outperformed salt tolerant variety Numar (2-fold vs 4.5-fold Gs reduction, respectively; both significant at P < 0.01). However, due to initially higher Gs values in control conditions for Numar, the difference in Gs between salt-grown GP and Numar genotypes was not statistically significant (at P < 0.05). Both these varieties retained significantly (P < 0.01) more water in the shoot compared with salt-sensitive NN (Figure 2D).

### Tissue ionic relations

**Figure 3:**
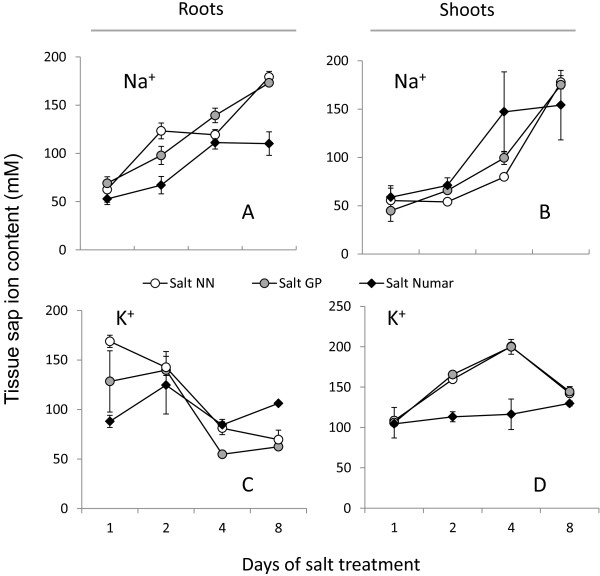
**Changes in root and shoot sap Na**^**+ **^**and K**^**+ **^**content in three barley cultivars contrasting in their salinity stress tolerance during salt stress progression.** Plants were grown hydroponically under control conditions until 3 days old, and then exposed to 100 mM NaCl treatment. Mean ± SE (n = 24).

Hydroponic experiments demonstrated that salt-tolerant Numar accumulated less Na^+^ in the root (Figure 3A) compared with two other cultivars, throughout the whole salt stress period. At the same time, Numar plants had twice as much Na^+^ in the shoot sap compared with other cultivar on day 4 (Figure 3B), however, similar shoot Na^+^ were observed between all three cultivars by day 8 (Figure 3B). There was no significant difference in either root or shoot Na^+^ accumulation between the three cultivars when grown in the absence of salt (data not shown).

Salinity stress resulted in a rapid and progressive decline in root K^+^ content in salt-sensitive cultivar NN (a 2-fold decrease from 128.4 ± 31 to 62.4 ± 2.7 mM over 8 days of 100 mM NaCl treatment; Figure 3C), while over the same time period, root K^+^ content did not change significantly in salt-tolerant cultivar Numar. The root K^+^ content in the intermediate salt tolerant GP also declined substantially, but not to the same extent as in NN (Figure 3C). Interestingly, while root K^+^ content dropped sharply in cultivars NN and GP, their shoot K^+^ content increased approximately 2 fold over the same time period (Figure 3D), suggesting a possible retranslocation of K^+^ from root to shoot. This observation was not seen for Numar (Figuer 3D). The mean values for root K^+^ content in controls were 111.2 ± 11.6, 153 ± 4.5 and 146.7 ± 1.9 mM, for NN, GP and Numar, respectively. As a result of better K^+^ retention and less Na^+^ accumulation, root Na^+^/K^+^ ratio was 2.5 fold higher in salt sensitive NN compared with tolerant Numar (2.57 vs 1.03, respectively; Figure 3).

### Salinity- and hydroxyl radical-induced ion flux kinetics

**Figure 4:**
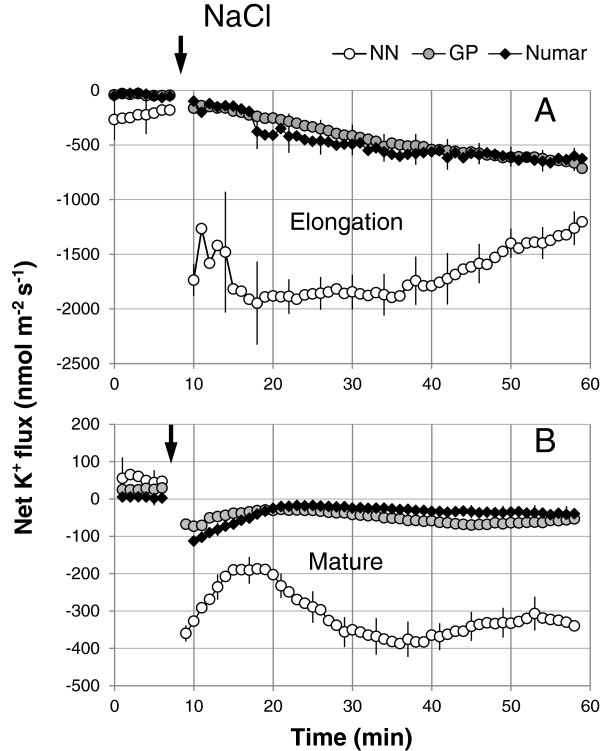
**Kinetics of NaCl- induced net K**^**+ **^**fluxes measured form the elongation (A) and mature (B) root zones of three barley cultivars in response to 100 mM NaCl treatment.** Mean ± SE (n = 6-8). The sign convention is “efflux negative”. The arrow indicates the application of the treatment.

Similar to our previous reports on barley [36,38,48], acute NaCl treatment induced massive K^+^ efflux from the epidermal cells of the root (Figure 4). Epidermal cells in the elongation zone had approximately 5-fold higher K^+^ efflux when compared with those in mature zone (Figures 4A and B, respectively; significant at P < 0.01). Responses of cultivar GP and Numar were very similar (no difference at P < 0.05) while the magnitude of NaCl-induced K^+^ leak from the roots of the salt-sensitive NN was approximately 4-fold greater than the response seen in the other two cultivars (Figure 4; significant at P < 0.05).

**Figure 5:**
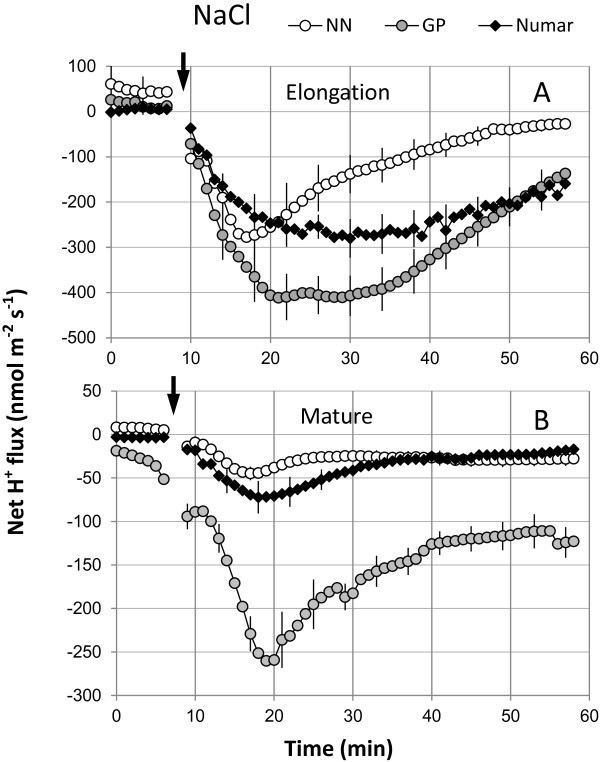
**Kinetics of NaCl- induced net H**^**+ **^**fluxes measured from the elongation (A) and mature (B) root zones of three barley cultivars in response to 100 mM NaCl treatment.** Mean ± SE (n = 6-8). The sign convention is “efflux negative”. The arrow indicates the application of the treatment.

Salinity treatment also induced a rapid (within one minute) net H^+^ efflux from both the elongation and mature root zones (Figure 5). This activation was strongest for GP, followed by Numar and then NN (Figure 5). The difference in H^+^ efflux amongst cultivars was significant at p < 0.01; and so was the difference between elongation and mature zones (Figures 5A and B). The NaCl-induced H^+^ efflux was more pronounced in the elongation zone in comparison to the mature zone.

**Figure 6:**
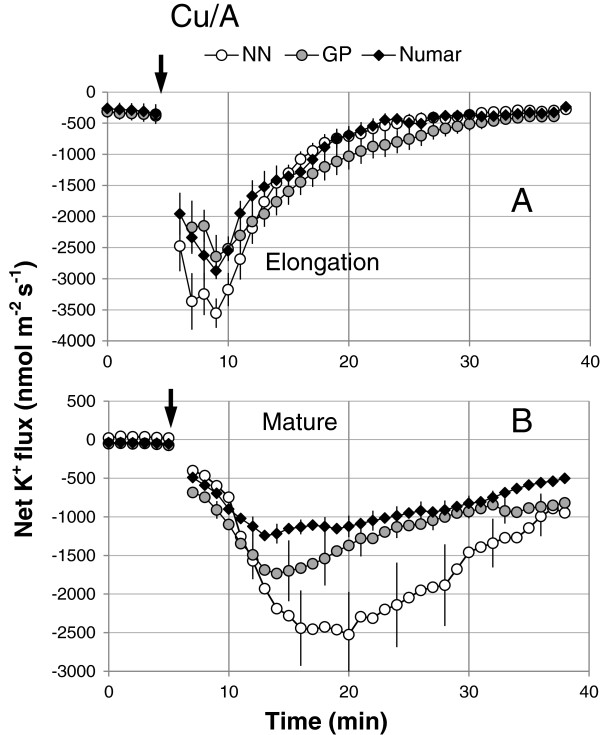
**Hydroxyl radical-induced changes in net K**^**+ **^**flux measured from the elongation (A) and mature (B) root zones of three barley cultivars.** The hydroxyl radical-generated copper ascorbate mix (0.3 mM CuCl_2_ and 1 mM Na^+^-Ascorbate) was added at the time indicated by an arrow. Mean ± SE (n = 6-8). The sign convention is “efflux negative”.

Consistent with previous observations [43,57], addition of hydroxyl radical-generating Cu/ascorbate mix also triggered massive K^+^ efflux from plant roots (Figure 6). Similar to NaCl stress, responses from elongation zone for each cultivar was significantly (P < 0.05) stronger compared with the mature root zone (Figure 6A and 6B, respectively). ROS induced K^+^ efflux was higher in the salinity sensitive cultivar NN followed by intermediate GP variety and then the tolerant cultivar Numar (Figure 6B; significant at P < 0.05).

### Transcriptional changes in the gene expression profile

**Figure 7:**
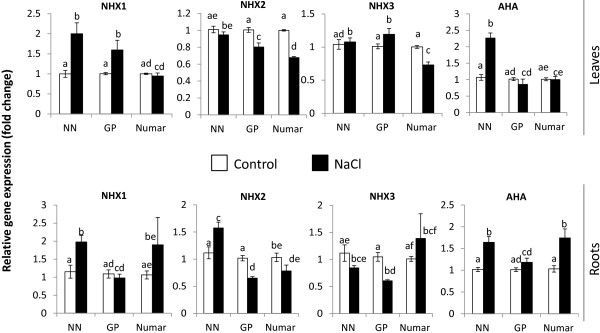
**Expression of barley *NHX1*, *NHX2*, *NHX3*, and *AHA2 *transporter genes in leaf and root tissues after 48 h of 100 mM NaCl treatment.** Mean ± SE (n = 12-15).

**Figure 8:**
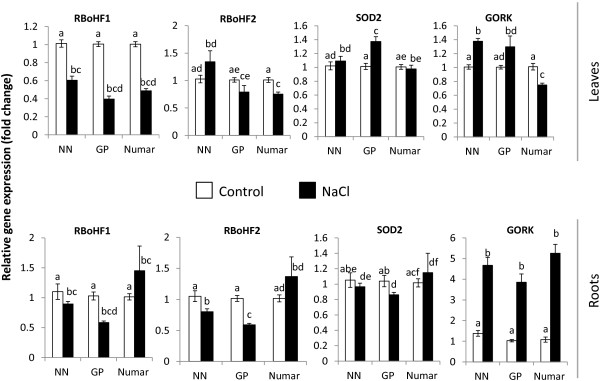
**Expression of barley *RBoHF1 *(Respiratory oxidative burst homologue/NADPH oxidase), *RBoHF2*, *SOD2 *and *GORK *genes in leaf and root tissues after 48 h of 100 mM NaCl treatment.** Mean ± SE (n = 12-15).

The expression of a number of key genes potentially involved in plant adaptive responses to salinity was examined in hydroponically-grown plants (Figures 7 and 8; Table 1). This included *NHX* (encoding tonoplast Na^+^/H^+^ exchanger and thus enabling vacuolar Na^+^ sequestration; [28-30,32,33]); *AHA* (confers activity of the P-type H^+^-ATPase and, thus, is critical for membrane potential maintenance and also “fuelling” of SOS1 and NHX Na^+^/H^+^ exchangers; [20]), and genes involved in ROS production (RBoHF, encoding NADPH oxidase, [58,59]) and detoxification (SOD, Superoxide dismutase [45,57]). The expression pattern of *GORK* was also examined, given the essential role cytosolic K^+^ retention plays in salinity tolerance in barley [36,38] with the GORK channel having a key role in this process [60]. All these genes were found to be the major contributors t plant salt tolerance in numerous previous studies [28-33,36,38,45,57,60,61].

**Table 1:** The relative gene expression of the gene studied in fold change presented as percentage

**Gene Name**	**% Fold change (p-value)**			**% Fold change (p-value)**		
	**Leaf**			**Root**		
	**Cultivar**			**Cultivar**		
	**NN**	**GP**	**Numar**	**NN**	**GP**	**Numar**
*Hv-NHX1*	200 (0.005)	160 (0.02)	−95 (NS)	171 (0.01)	−89 (NS)	178 (NS)
*Hv-NHX2*	−94 (NS)	−80 (0.0001)	−68 (0.0001)	141 (0.03)	−64 (0.0001)	−76 (NS)
*Hv-NHX3*	104 (NS)	118 (0.01)	−73 (0.0009)	−76 (NS)	−58 (0.0001)	137 (NS)
*Hv-RBoHF1*	−60 (0.0001)	−39 (0.0001)	−49 (0.0001)	−81 (NS)	−57 (0.0001)	143 (NS)
*Hv-RBoHF2*	131 (NS)	−78 (NS)	−74 (0.0001)	−76 (0.05)	−58 (0.0001)	134 (NS)
*Hv-PMHATPase*	212 (0.0001)	−84 (NS)	99 (NS)	161 (0.0005)	117 (NS)	169 (0.02)
*Hv-SOD2*	107 (NS)	136 (0.0001)	97 (NS)	−92 (NS)	−83 (0.05)	113 (NS)
*Hv-GORK*	137 (0.006)	129 (NS)	−74 (0.0001)	339 (0.0001)	375 (0.0001)	487 (0.0001)

NS-Non-significant; − sign indicate down-regulation.

As shown in Figures 7 and 8, there was a large range in the transcriptional response in the eight genes studied to salt application in both leaves and roots. However no clear patterns of gene expression could be observed at this specific time point which could explain the difference in tolerance between the three cultivars. Of three homologues to the *AtNHX* gene, which was originally suggested to encode a protein involved in Na^+^ sequestration into the vacuoles, a 2-fold increase in *NHX1* transcript level was measured both in the leaf and root of the salt sensitive NN (Figure 7). No significant changes in *NHX1* expression were measured in the leaf and root of the salt-tolerant Numar plants, while in the intermediate GP significant *NHX1* upregulation was only observed in the leaves (Figure 7). *NHX2* transcript levels went down in both leaves and roots in cultivars Numar and GP but not in salt-sensitive NN (Figure 7), while no clear patterns were observed for *NHX3* transcripts.

During salinity treatment there was a relative increase in the expression of the barley homologue *AHA2* in the roots of both Numar and NN, while a significant induction was observed only in the leaves of NN (Figure 7). No significant (P < 0.05) changes in *AHA2* transcript levels were detected in GP plants.

The relative gene expression of the barley homologue of *RBoHF1*, important in encoding for NADPH oxidase was down-regulated significantly (P < 0.01) by about 2-fold in the leaves of all the cultivars after salt application (Figure 8), however, no clear patterns in the roots were detected. *FBoHF2*, also involved in the production of NADPH oxidase was only slightly down-regulated after salt application (significant for Numar in leaves and NN and GP in roots; Figure 8). The barley homologue of *SOD2* relative gene expression showed small increase in leaves of GP cultivar but no significant changes for any other varieties in either tissue (Figure 8).

The largest and most striking difference was observed for NaCl-induced changes in the relative transcript abundance of the barley homologue of *GORK* in barley roots, with 4 to 5-fold increase reported for all cultivars (Figure 8; all significant at P < 0.01). In leaves, small but significant (at P < 0.05) increase in *GORK* transcript level was measured in the salt-sensitive NN while the *GORK* transcripts in the leaf of salt-tolerant Numar were reduced (Figure 8).

## Discussion

### No obvious relationship between gene expression in seedlings and overall barley performance under saline conditions

Vacuolar sodium sequestration is essential to avoid Na^+^ cytotoxicity, and increased activity of *NHX* tonoplast Na^+^/H^+^ exchangers was shown to enhance salinity tolerance in plants [29,62]. However, in the work presented here a 2-fold increase in *NHX1* transcript level was measured in both leaves and roots of salt-sensitive NN (Figure 7), while no significant (at P < 0.05) changes were measured in salt-tolerant Numar plants in either tissue. Consistent with this, *NHX2* transcript levels went down in both tissues in cultivars Numar and GP but not in salt-sensitive NN (Figure 7). Thus, it appears that at the measured time point changes in transcript levels of the *NHX*s cannot be used to predict the salinity stress tolerance of barley in our experiments.

Several possible explanations should be considered for these observations. First, as described above, there is recent evidence that the major role of *AtNHX1* may be in K^+^ but not Na^+^ transport [34], thus the genes studied here may not encode proteins involved in Na^+^ compartmentation. Second, the higher NaCl induced expression of *NHX*s in the shoots of the salt-sensitive NN could be to compensate for its inability to prevent Na^+^ delivery to the shoot (e.g. a need to deal with the consequences of cytosolic Na^+^ accumulation). However, this explanation is unlikely as the shoot Na^+^ content of the salt tolerant Numar was higher than that of NN between days 2 and 4 of salt stress (the time when gene expression of the *NHXs* were assessed). Another explanation is that post-translational modifications such as protein folding are much more important that gene expression to have a proper functional response – the proteins are already present at desired concentrations (therefore gene expression is not required), however, need to be activated by post-translational modifications, a process which allows a plant to respond faster to stress than relying solely on gene expression. It should also be noted that only one time point was used for the expression studies, and expression of genes encoding salt tolerance gene has been shown to fluctuate between days (e.g. *HVP10* in [63]). Lastly, it should also be noted the *NHX1* and *NHX2* genes in barley are homologues of *AtNHX1* and, while having similar nucleotide sequence may not necessarily confer the protein(s) function as AtNHX1 in Arabidopsis.

The above notion that measuring the changes in transcript levels at one time point early on during seedling growth does not allow the ability to predict barley salinity stress tolerance is further corroborated by the study of plant oxidative stress responses. No significant difference in the relative expressions of *RBoHF1* and *RBoHF2* which encode the NADPH oxidase/Nox (one of the major sources of ROS production under saline conditions; [58,59]) were found in roots of the salt sensitive NN and salt tolerant Numar cultivars (Figure 8). Similarly, salt stress did not result in any significant changes in *SOD* expression level in either root or leaf tissues of these varieties (Figure 8). At the same time, NN roots were twice more sensitive to ROS treatment (Figure 6B).

Other evidence comes from comparing the NaCl-induced net H^+^ fluxes measured in barley roots (Figure 5) with the changes in the *AHA* (encoding plasma membrane H^+^-ATPase) transcript levels (Figure 7). H^+^-ATPase activity is indispensable for maintaining membrane potential (MG Palmgren and P Nissen [20], and intrinsically higher H^+^-ATPase activity was shown to correlate with salinity tolerance in barley [38]. In this study, the GP cultivar showed consistently higher proton efflux in both elongation and mature zone compared with two other cultivars under hydroponic conditions (Figure 5A and 5B). However, the expression of the *AHA* barley homologue was shown to be lower compared to the other two cultivars suggesting that at this time point H^+^ pumping activity may be higher in GP cultivar.

It should be also added that plant responses to salinity stress may differ dramatically between hydroponics and soil systems [64]. While soils are extremely complex and heterogeneous media, with pronounced physical, chemical, and biological gradients observed in root rhizosphere, ionic conditions around roots are more or less uniform in hydroponics. Specifically, no depletion zones will be present in the latter case; salinity build-up in the rhizosphere will be also prevented by the continuous solution mixing. Thus, to properly adapt to such conditions plant roots may require a different set of transporters as compared with the soil system. Also different may be the gene expression patterns.

### Plant tissue tolerance was a dominating component that has determined the overall plant responses to salinity

Salinity tolerance is a physiologically multi-faceted trait attributed to multiple mechanisms; these can be roughly divided into ionic, osmotic and oxidative components [6]. While all of them are indeed important, their relative contribution may differ, depending on plant species, stress severity and duration, and experimental conditions. In this work we have attempted to quantify the relative contribution of each of these components towards the overall salinity stress tolerance in barley.

The tolerant cultivar Numar maintained root K^+^ content at a constant level throughout the eight day salt stress period, while the two other varieties showed a progressive decline in root K^+^ content (with the greatest decline in the salt-sensitive NN) (Figure 3C). This was further corroborated in MIFE experiments measuring NaCl-induced K^+^ efflux from barley roots (Figure 4). The rationale behind this experiment is that when Na^+^ is absorbed from extracellular space, the membrane gets depolarised and this depolarisation of cell membrane initiates potassium leak as a result of the activation of depolarisation activated outward-rectified potassium channels (KOR) [38,65]. This reduces the K^+^ content in the cytosol negatively affecting cell metabolism [60] and in turn brings about programmed cell death [43,66]. Potassium loss from the epidermal cell in the elongation and mature zone of three barley plants was studied and the highest potassium loss was observed in the salt sensitive cultivar NN followed by the tolerant cultivars (Numar and GP) in both zones (Figure 4A and B), reflecting overall tolerance estimated by agronomical (biomass accumulation; Figure 1) and whole-plant physiological (chlorophyll content; Figure 2) characteristics. At the same time, osmotolerance appears to be not central to the overall plant performance under saline conditions. Indeed, the intermediate salt tolerant GP outperformed the salt-tolerant cultivar Numar, having highest Gs and shoot water content values under saline conditions (Figure 2). This corroborates the point made by K Rajendran, M Tester and SJ Roy [49] that superiority in one salt tolerant component does not guarantee an enhanced overall salt tolerance performance.

Gas exchange (Gs) could be considered as a yield determinant and a valuable tool as a physiological trait that can readily be used as a breeding tool [67,68]. Carbon entry and transpiration occurs using open stomata which both helps to increase photosynthesis and nutrient absorption from the growth media. However, under saline conditions, the stomata tend to close to prevent water loss as it is a scarce resource due to osmotic imbalance. Thus, higher Gs values will ensure better CO_2_ assimilation ability (and, hence, higher yield) only when plants have a biochemical and physiological capacity to fully utilise it. This means that higher Gs values must be complemented by higher shoot tissue tolerance, to ensure efficient leaf photochemistry under saline conditions.

To our great surprise, the expression of the barley homologue of *GORK* gene showed high up-regulation in all cultivars in our hydroponics assay. This is counterintuitive, as the opposite effect would be hypothesised due to the very strong correlation between barley salinity stress tolerance and its ability to prevent NaCl-induced K^+^ leak from roots [36,38,39]. These findings, however, may be due to the differences between the experimental systems used or due to the notion that transcriptional changes appear to be causally unrelated to functional plant responses. It could be suggested that the post-translational phenomena of assembly might play a role in the regulation of this gene as it is fundamental for the pore formation and electric activity to form tetramer of its α-subunit [69]. Furthermore, the availability of the α-subunits and its functional tetrameric assembly is of paramount importance for the cell [70].

Another fact supporting the essential role of tissue tolerance in overall plant performance under saline conditions could be found in kinetics of Na^+^ accumulation in the shoot (Figure 3). Consistent with previous reports [26,27,47], the tolerant cultivar Numar tends to send more Na^+^ to the shoot via the transpiration stream and use it as a cheap osmoticum to maintain shoot turgor, while sensitive variety NN delayed this process favouring Na^+^ accumulation in the roots (Figure 3A and B). Despite this, Numar plants were able to maintain higher chlorophyll levels while massive chlorosis was observed in NN variety (Figure 3). As the volume of the leaf epidermis is small; the explanation for Na^+^ sequestration would be vacuolar compartmentalisation in the mesophyll cells. Consistent with this, salinity treatment caused an increase in the size of palisade parenchyma cells; this increase was much higher in the tolerant barley cultivars [26]. A similar phenomena is widespread in halophytes [4], species considered to be most tolerant to salt stress. Halophytes also use additional tissue tolerance mechanism such as salt bladders to accumulate excess Na^+^ away from the photosynthetic tissue.

### Hydroxyl radical-induced K^+^ loss is negatively correlated with salinity stress tolerance

In addition to the two above components of the tissue tolerance mechanism, namely better K^+^ retention by roots and more efficient vacuolar Na^+^ sequestration in shoots, salinity stress tolerance in barley is also correlated strongly with its ability to prevent hydroxyl radical/induced K^+^ loss (Figure 6). The highest potassium efflux was observed from the salt sensitive cultivar NN, followed by GP (intermediate) and Numar (tolerant). The hydroxyl radical-induced effects were higher in the mature root zone (Figure 6A and B). These findings are consistent with previous reports for root sensitivity to H_2_O_2_[38,48] suggesting that sensitivity to oxidative stress is an essential component of the tissue tolerance mechanism.

An increase in ROS production under salinity stress is attributable to activity of NADPH oxidase, a cell wall-associated peroxidase that generates O_2_·- by oxidizing NADPH and transferring the electron to oxygen (O_2_) [61]. Accordingly, the regulation of the gene at the transcript level was studied. The relative gene expression at the transcript level of the barley homologue *RBoHF* gene for all of the cultivars was significantly down-regulated but only in leaves but not roots (Figure 8). This down-regulation was least in salt-sensitive NN, suggesting a possible causal link between NADPH oxidase activity and tissue tolerance mechanisms in salinised mesophyll tissues. However, unlike the leaf, in the root the transcript abundance was non-significant, except for GP (Figure 8).

Potassium efflux induced by hydroxyl radicals is mediated by two major transport systems: (i) non-selective cation channel (NSCC) and (ii) depolarization-activated K^+^-selective outward rectifying channel [43,71]. High cytosolic K^+^ levels are also essential to suppress activity of caspase-like proteases and endonucleases, both in mammalian [72,73] and plant [42,43] systems. Decrease in the cytosolic K^+^ pool in plant roots may result in activation of these catabolic enzymes triggering programmed cell death (PCD), especially in sensitive root apex cells (Figure 6).

## Conclusion

In this study, we show that much of the genes that have been known to encode key physiological mechanisms conferring plant salt stress tolerance have lower explanatory value towards the contribution of this gene towards plants adaptive responses to salinity, when assessed at the transcriptional level. This was likely to do with focusing on the transcriptional response at a particular time point that may not necessarily reflect the overall plant performance during the life span of experiment. Post-translational (e.g. functional) modifications may be another reason to explain this apparent controversy. For better tissue tolerance, sodium sequestration, K^+^ retention and resistance to oxidative stress all appeared to be crucial. These traits seem to be highly interrelated, as cytosolic K^+^ retention is essential for the optimal vacuolar H^+^ pump operations [4] required to fuel *NHX* Na^+^/H^+^ exchanger to enable Na^+^ sequestration. Cytosolic K^+^ homeostasis, in its turn, is strongly affected by the sensitivity of plasma membrane transporters to ROS and the plant’s ability to prevent stress-induced hydroxyl radical accumulation in stressed tissues. Thus, it appears that major progress in crop breeding for salinity tolerance can be achieved only if these complementary traits are targeted at the same time.

## Competing interests

The authors declare that they have no competing interests.

## Author’s contributions

GA conducted the bulk of experiments and wrote the paper draft. SS was responsible for experimental design and data interpretation, and took the leading role in writing. SR was leading the transcriptomic work, critically assessed all the data and commented on the manuscript. JB and MZ have contributed to data analysis and paper writing and provided a logistical support for this work. All authors read and approved the final manuscript.

## Supplementary Material

Additional file 1: Table S1 The Primers used in the gene expression study and their respective amplicon size. Click here for file
