# Supplementary material for: Evaluating contribution of ionic, osmotic and oxidative stress components towards salinity tolerance in barley
Source: BMC Plant Biol. 2014 Apr 28;14:113. doi: 10.1186/1471-2229-14-113 (PMC4021550; doi:10.1186/1471-2229-14-113)
Supplement: Additional file 1: Table S1 — The Primers used in the gene expression study and their respective amplicon size. [file 1471-2229-14-113-S1.doc]

Table S1 The Primers used in the gene expression study and their respective amplicon size.

| **Gene Name** | **Forward Primer** | **Reverse Primer** | **Amplicon Size (bp)** |
| --- | --- | --- | --- |
| *Hv-NHX1* | TGCATATCTACCAGTGCTTAT | GGTTCAAGACACAAGTTCAGT | 184 |
| *Hv-NHX2* | GGTTTTCGGCTTGCTGACTAA | CATTGGGCGCATGAACTTATC | 238 |
| *Hv-NHX3* | TGAGCCGAACATTACTGTGAT | ACGAGCTTACCTTTCAATACA | 127 |
| *Hv-RBoHF1* | TTACAACATGGACCTGCGTCCCTACA | TGCCTTGGTCAGACACTCAGCTGCAT | 206 |
| *Hv-RBoHF2* | TATGCGGAGTCCCGCAGAAAGATG | TGTACTGTACTCCCCCTGCCTGTGT | 212 |
| *Hv-PMHATPase* | CTTGGTTATCGCCTTCCTTC | GCCACTCAGCACAAATCG | 184 |
| *Hv-SOD2* | CTTGAAGGACACCGACTTGC | CTCAAAAAGCCAAATGACAGTG | 140 |
| *Hv-GORK* | CCACACGAGGCGAAGAAG | GAGGAATCCACAGCATCACC | 194 |
| *Hv-GAPdH2* | GTGAGGCTGGTGCTGATTACG | TGGTGCAGCTAGCATTTGAGAC | Control gene |
